# Remarkable increase in Interleukin-5 receptor expression beyond tissue eosinophils in inflammatory bowel disease

**DOI:** 10.3389/fimmu.2025.1589421

**Published:** 2025-05-22

**Authors:** You Ie Kim, Hyeon Jeong Oh, Hye Ran Yang

**Affiliations:** ^1^ Department of Pediatrics, Seoul National University College of Medicine, Seoul, Republic of Korea; ^2^ Department of Pediatrics, Incheon St. Mary’s Hospital, College of Medicine, The Catholic University of Korea, Seoul, Republic of Korea; ^3^ Department of Pathology, Seoul National University Bundang Hospital, Seongnam, Republic of Korea; ^4^ Department of Pediatrics, Seoul National University Bundang Hospital, Seongnam, Republic of Korea

**Keywords:** eosinophil, interleukin-5 receptor, inflammatory bowel disease, pathogenesis, proinflammatory cytokine

## Abstract

**Introduction:**

The role of tissue eosinophils and their relationship with interleukin-5 in gastrointestinal disorders have not been elucidated. This study aimed to analyze tissue eosinophils and interleukin-5 receptor-α subunit (IL-5RA) expression on eosinophils in inflammatory bowel disease (IBD).

**Methods:**

Blood and stool test results and radiologic and endoscopic findings in pediatric patients, who were newly diagnosed with IBD, eosinophilic gastrointestinal diseases (EGIDs), or disorders of gut–brain interactions were retrospectively reviewed. Tissue eosinophil counts were analyzed, and immunohistochemistry was performed using an antibody against IL-5RA expressed on eosinophils in the colonic mucosa.

**Results:**

The tissue eosinophil count and IL-5RA-expressing eosinophil density were significantly different among the three groups (*P*<0.005). IL-5RA expression was significantly higher in the IBD group than in the EGID group (*P*<0.001). In IBD, tissue IL-5RA expression was positively correlated with platelet count, erythrocyte sedimentation rate, and highly sensitive C-reactive protein, and negatively correlated with hemoglobin and albumin. In Crohn’s disease (CD), higher IL-5RA expressions were observed with higher pediatric CD activity index (*P*<0.005). The optimal cut-off value for IL-5RA-expressing eosinophil density was 69.4/high-power field.

**Discussion:**

The expression of IL-5RA in tissue eosinophils is elevated in treatment naïve IBD. IL-5RA expression may reflect disease severity and its extent of involvement in early active CD.

## 1 Introduction

Inflammatory bowel disease (IBD) is caused by various factors, such as genetics, environmental factors, the microbiome, and host immunity. Both innate and adaptive immune cells are involved (1, 2), along with proinflammatory cytokines, such as tumor necrosis factor-α and interleukin (IL) -6, -12, -23, -17, and -18 secreted by these immune cells (3).

Eosinophils, primarily active in allergic reactions and parasitic infections (4), have been linked to gut inflammation (5). In addition to eosinophilic gastrointestinal (GI) diseases (EGIDs), characterized by eosinophil infiltration in the gut, disorders of gut–brain interactions (DGBI) involve eosinophils and other immune cells (6). Furthermore, some reports have suggested that eosinophils are involved in IBD pathogenesis (7–10).

IL-5, a key proinflammatory cytokine that affects the differentiation, maturation, migration, and tissue recruitment of eosinophils (11), is secreted by eosinophils, mast cells, and CD4+ and CD8+ T cells (8, 12–14). Secreted IL-5 induces the expression of Janus kinase (JAK) binding proteins by binding to the IL-5 receptor-α subunit (IL-5RA) on intestinal mucosal tissue eosinophils (11, 15). The JAK and signal transducer and activator transcription pathways are involved in intestinal homeostasis and inflammation, playing a crucial role in IBD pathogenesis and treatment (16). However, a clear relationship among eosinophils, IL-5, and IBD has not yet been established (14).

Therefore, this study aimed to identify tissue eosinophils and IL-5RA expression in IBD and to elucidate their role in IBD pathogenesis compared to EGID and DGBI. We analyzed and compared immunohistochemical staining for IL-5RA, which is specifically expressed in eosinophils, in the colonic tissues of pediatric patients newly diagnosed with IBD, EGID, or DGBI.

## 2 Materials and methods

### 2.1 Patients and diagnostic criteria

This study included pediatric patients of <18 years of age who were diagnosed with IBD, EGID, or DGBI at the Seoul National University Bundang Hospital between May 2003 and August 2023. During this period, patients presenting with chronic GI symptoms underwent blood and stool tests, imaging studies, endoscopy, and tissue biopsies of the GI tract. Based on the results, the enrolled patients were classified as follows:

IBD was diagnosed and classified into three subgroups, Crohn’s disease (CD), ulcerative colitis (UC), and IBD-unclassified (IBD-U), according to the revised Porto criteria in 2014 (17).

Based on the extent of tissue eosinophilic infiltration, EGID was classified into subgroups, including eosinophilic esophagitis, eosinophilic gastritis, eosinophilic enteritis, and eosinophilic colitis (18, 19). This study focused on patients diagnosed with eosinophilic colitis, defined as eosinophilic infiltration exceeding 50 per high-power field in the right colon, 35 in the transverse colon, and 25 in the left colon and rectum (18–23).

DGBI, previously known as a functional abdominal pain disorder, represents a complex spectrum of GI disorders characterized by chronic or recurrent symptoms without apparent organ pathology. During the study period, the enrolled patients were diagnosed with DGBI according to the Rome IV criteria (24, 25).

All patients included in this study were newly diagnosed based on the diagnostic criteria at initial visit to our tertiary hospital. Among these patients, only those who voluntarily provided informed consent for IL-5RA immunohistochemical staining were enrolled in the study. Patients diagnosed with other organ diseases as well as those who had a recent GI tract infection, were excluded. Additionally, all enrolled patients were not prescribed anti-inflammatory medication.

### 2.2 Laboratory, radiologic, and endoscopic findings

Data on blood and stool tests, imaging studies, and endoscopic findings were retrospectively reviewed. In the blood tests, complete blood count, erythrocyte sedimentation rate (ESR), and highly sensitive C-reactive protein (hsCRP) levels, which are indicators of inflammation in the blood, were evaluated. Additionally, data on total immunoglobulin E and eosinophil cationic protein (Phadia AB, Sweden fluorescent enzyme immunoassay), which are related to allergies, were collected. Albumin, which can represent absorption in the GI tract, was analyzed. Fecal calprotectin, an indicator of GI inflammation in the stool, was measured using fluorescence enzyme immunoassay (Calprotectin, Phadia AB, Sweden).

The endoscopic findings revealed the depth and location of the mucosal lesions. Endoscopic findings were collected using the Simple endoscopic score for CD (SES-CD) and the Mayo score for UC. Additionally, small bowel lesions, such as stricture, penetration, and perianal abscess and fistula, were evaluated using magnetic resonance enterography. Based on magnetic resonance enterography and endoscopic findings, disease status was determined using the Paris classification in patients with IBD of <18 years of age (26). Disease severity was measured using the pediatric Crohn’s disease activity index (PCDAI) in CD and pediatric ulcerative colitis activity index in UC (27, 28).

### 2.3 Histopathologic findings

During colonoscopy, biopsies were performed on the cecum, ascending colon, transverse colon, descending colon, sigmoid colon, and rectum, regardless of the presence of lesions. The tissue samples from two colon biopsies were obtained from each patient. For IBD and EGID, tissues were selected from a segment with a lesion or high eosinophil infiltration. For DGBI, tissues were selected randomly.

First, to count the tissue eosinophils, the tissues were fixed in formalin immediately after endoscopic biopsy, embedded in paraffin wax, cut into 3-μm slices, and stained with hematoxylin and eosin. Histopathologic findings and high-magnification eosinophil infiltration were examined by two pathologists at the Pathology Department of Seoul National University Bundang Hospital using an electron microscope.

Second, for the same tissues, immunohistochemical staining was performed on unstained slides to identify IL-5RA expression in eosinophils. Anti-IL-5RA antibody [CAL40] (Abcam, Cambridge, UK) was used for immunohistochemical staining with the Roche BenchMark ULTRA IHC/ISH Staining Module (Ventana Medical Systems, Tucson, AZ, USA) according to the standard operating procedure at the SuperBioChips Laboratory, Seoul, South Korea.

Immunohistochemical staining showed that the anti-IL-5RA antibody darkly stained the eosinophils in the colonic mucosa, whereas other types of cells were not stained. Five high-power fields with a high density of positive cells, excluding lymphoid follicles and erosions, were selected by a pathologist, and the density of positive cells in each high-power field was assessed with image analyzer-assisted measurement automatically. The number of positive cells per 0.237 mm^2^ was measured to calculate the density of positive cells.

### 2.4 Ethics approval and consent to participate

The study was approved by the Institutional Review Board of Seoul National University Bundang Hospital (No. B-2310-860-302), and informed consent was obtained from the patients and legal representatives of children of 6–18 years of age. This study was performed in accordance with the Declaration of Helsinki and approved guidelines and regulations.

### 2.5 Statistical analyses

Data were analyzed using SPSS Statistics version 26.0 (IBM Corporation, New York, NY, USA). Results are presented as median (range) for parametric data, with one-way analysis of variance applied to non-parametric statistics, followed by Bonferroni correction for *post hoc* analysis. For continuous variables, the Kruskal–Wallis and Mann–Whitney tests were employed. Categorical variables, described as number (%), were analyzed using Fisher’s exact test. Pearson and Spearman’s correlation analyses were conducted to assess the correlation between IL-5RA and other variables.

Diagnostic accuracy and optimal cut-off values were determined using the area under the receiver-operating characteristic curve (AUROC) with a 95% confidence interval. Significance was set at P <0.05.

## 3 Results

### 3.1 Patient characteristics

A total of 81 patients were enrolled: 50 with IBD, 21 with EGID, and 10 with DGBI. Of the patients with IBD, 23 (46%) had CD, 21 (42%) UC, and 6 (12%) IBD-U.

Basic demographic characteristics and laboratory findings are described in **Table 1**. Laboratory results for inflammation, such as absolute neutrophil count, hemoglobin, platelet, ESR, hsCRP, albumin, and fecal calprotectin levels, showed significant differences among the three groups.

**Table 1:** Comparison of demographic features and laboratory findings among children with inflammatory bowel disease, eosinophilic gastrointestinal diseases, and disorders of gut–brain interaction.

Variable	IBD (n=50)	EGID (n=21)	DGBI (n=10)	*P-*value (total)	*P-*value (IBD-EGID)	*P-*value (EGID- DGBI)	*P-*value (IBD- DGBI)
Male, n (%)	33 (66.0%)	13 (61.9%)	5 (50.0%)	**0.020**	0.789	0.530	0.338
Age, years, median (range)	14.5 (7.0–17.7)	12.1 (3.1–16.9)	13.8 (5.1–17.1)	0.171	0.253	0.854	0.946
Past history of allergic disease, n (%)	15 (30.0%)	16 (76.2%)	4 (40.0%)	0.222	**<0.001**	**0.049**	0.535
Laboratory findings, median (range)							
WBCs (/μL)	8,840 (4,950–33,420)	7,065 (3,670–10,860)	6,910 (5,810–8,730)	0.530	**0.029**	0.998	**0.011**
ANC (/μL)	5,251 (1,775–24,407)	3,269 (1,410–8,257)	3,036 (1,685–5,439)	**0.014**	**0.011**	0.594	**<0.001**
AEC (/μL)	189 (23–1,018)	183 (48–3,258)	155 (90–384)	0.443	0.894	0.568	0.161
Hemoglobin (g/dL)	13.0 (4.2–15.8)	13.3 (11.9–16.2)	13.0 (11.5–15.4)	**0.018**	**0.009**	0.841	0.196
Platelets (/μL)	359 k (189 k–821 k)	283 k (166 k–453 k)	283 k (188 k–384 k)	**<0.001**	**0.003**	0.609	**<0.001**
ESR (mm/hr)	11 (2–120)	5 (2–16)	3 (2–12)	**<0.001**	**<0.001**	0.466	**<0.001**
hsCRP (mg/dL)	0.22 (0.01–14.7)	0.03 (0.01–0.40)	0.02 (0.01–0.49)	**0.001**	**0.027**	1.000	0.124
Serum albumin (g/dL)	4.2 (2.6–5.2)	4.5 (3.9–5.3)	4.5 (4.3–4.9)	**<0.001**	**<0.001**	1.000	**<0.001**
Fecal calprotectin(mg/kg)	2,501 (4.6–6,000)	113.5 (6.2–2,155)	98 (3.5–993)	**<0.001**	**<0.001**	0.982	**<0.001**
Total IgE (IU/mL)	178.9 (1.7–2,500)	337.5 (20–2,035)	97.2 (3.9–622.4)	0.078	0.296	0.056	0.456
Serum ECP (μg/L)	25.9 (5.3–110.0)	33.2 (4.8–93.0)	14.6 (2.9–42.0)	0.117	0.980	**0.047**	**0.046**
Peripheral eosinophilia, n (%)	3 (6.0)	1 (4.8)	0 (0.0)	<0.001	0.836	0.483	0.427

*P*–values of < 0.05 indicate statistical significance. *P*–values indicating statistical significance highlight in bold for clarity.

IBD, inflammatory bowel disease; EGID, eosinophilic gastrointestinal disease; DGBI, disorder of gut–brain interaction; WBC, white blood cell; ANC, absolute neutrophil count; AEC, absolute eosinophil count; ESR, erythrocyte sedimentation rate; hsCRP, highly sensitive C-reactive protein; IgE, immunoglobulin E; ECP, eosinophil cationic protein.

The characteristics of the pediatric IBD subtypes are described in **Table 2**. According to the PCDAI, mild, moderate, and severe CD were observed in 11 (47.8%), 9 (39.1%), and 3 (13.0%) patients, respectively, and according to the pediatric ulcerative colitis activity index, mild, moderate, and severe UC were observed in 8 (38.1%), 7 (33.3%), and 6 (28.6%) patients, respectively.

**Table 2:** Comparison of clinical features and laboratory findings among subtypes of pediatric inflammatory bowel disease.

Variable	CD (n=23)	UC (n=21)	IBD-U (n=6)	*P-*value (total)	*P-*value (CD*-*UC)	*P-*value (CD*-* IBD-U)	*P-*value (UC*-* IBD-U)
Male, n (%)	16 (69.6%)	14 (66.7%)	3 (50%)	**0.024**	0.837	0.369	0.456
Age, years, median (range)	14.4 (8.0–16.7)	14.3 (7.0–16.9)	16.4 (8.5–17.7)	0.312	0.733	0.158	0.195
History of allergic disease, n (%)	9 (39.1%)	5 (23.8%)	1 (16.7%)	**0.005**	0.312	0.234	0.711
PCDAI or PUCAI, median(range)	30.0 (5.0 – 67.5)	50.0 (5.0–85.0)	PCDAI 27.5 (10.0–62.5) PUCAI 37.5 (20.0–75.0)			0.773	0.932
Paris classification (n, %)							
Age at diagnosis							
A1a (0–<10 y)	5 (21.7)		1 (16.7)				
A1b (10–<17 y)	18 (78.3)		2 (33.3)				
A2 (17–40 y)			3 (50.0)				
Location							
L1 (Ileal)	10 (43.5)		1 (16.7)				
L2 (Colonic)	5 (21.7)		2 (33.3)				
L3 (Ileocolonic)	8 (34.8)		3 (50.0)				
Behavior							
B1 (Non-stricturing/non-penetrating)	18 (78.3)		2 (33.3)				
B2 (structuring)	4 (17.4)		4 (66.7)				
B3 (Penetrating)	1 (4.3)						
P (perianal disease)	12 (52.2)		0 (0.0)				
G (growth delay)	1 (4.3)		1 (16.7)				
Extent							
E1 (Proctitis)		5 (23.8)					
E2 (Left-sided)		3 (14.3)	1 (16.7)				
E3 (Extensive)		2 (9.5)					
E4 (Pancolitis)		11 (52.4)	5 (83.3)				
**Severe**		6 (28.6)	2 (33.3)				
Laboratory findings, mean ± SD							
WBCs (/μL)	7,960 (4,600–15,200)	8,380 (4,950–17,450)	10,220 (4,780 – 33,420)	0.637	0.805	0.477	0.345
ANC (/μL)	5,107 (1,955–9,624)	5,208 (1,775–15,286)	6,764 (2,868–28,407)	0.588	0.549	0.581	0.345
AEC (/μL)	178 (23–1,018)	161 (0–477)	261 (19–752)	0.612	0.581	0.445	0.441
Hemoglobin (g/dL)	13.1 (8.5–15.2)	13.0 (4.2–15.8)	9.9 (8.6–13.5)	0.103	0.541	0.054	0.057
Platelets (/μL)	367 k (189 k–801 k)	359 k (211 k–637 k)	417 k (283 k–821 k)	0.251	0.385	0.302	0.110
ESR (mm/hr)	22 (2–120)	11 (2–48)	35 (2–110)	0.317	0.184	0.854	0.263
hsCRP (mg/dL)	1.04 (0.01–15.9)	0.15 (0.01–14.7)	0.5 (0.01–3.89)	**0.029**	**0.009**	0.328	0.441
Serum albumin (g/dL)	4.2 (2.8–5.0)	4.4 (2.7–5.2)	3.5 (2.6–4.4)	**0.039**	0.403	**0.036**	**0.012**
Fecal calprotectin (mg/kg)	1,466 (4.6–6,000)	2,129 (261–6,000)	3,684 (2,000–6,000)	**0.034**	0.088	**0.031**	0.095
Total IgE (IU/mL)	178.9 (1.7–2,500)	184.7 (26.9–1,022)	79.7 (58.9–274.7)	0.708	0.971	0.462	0.494
Serum ECP (μg/L)	26.2 (6.0–110.0)	27.5 (5.3–82.4)	14.6 (5.5–48.8)	0.638	0.755	0.495	0.391
Peripheral eosinophilia, n (%)	2 (8.7%)	0 (0.0)	1 (16.7%)	<0.001	0.323	0.965	0.057

*P*-values of < 0.05 indicate statistical significance. *P*–values indicating statistical significance highlight in bold for clarity.

CD, Crohn’s disease; UC, ulcerative colitis; IBD-U, inflammatory bowel disease-unclassified; PCDAI, pediatric Crohn’s disease activity index; PUCAI, pediatric ulcerative colitis activity index; SD, standard deviation; WBC, white blood cell; ANC, absolute neutrophil count; AEC, absolute eosinophil count; ESR, erythrocyte sedimentation rate; hsCRP, highly sensitive C-reactive protein; IgE, immunoglobulin E; ECP, eosinophil cationic protein.

### 3.2 Tissue eosinophil count and IL-5RA analysis

**Figure 1:**
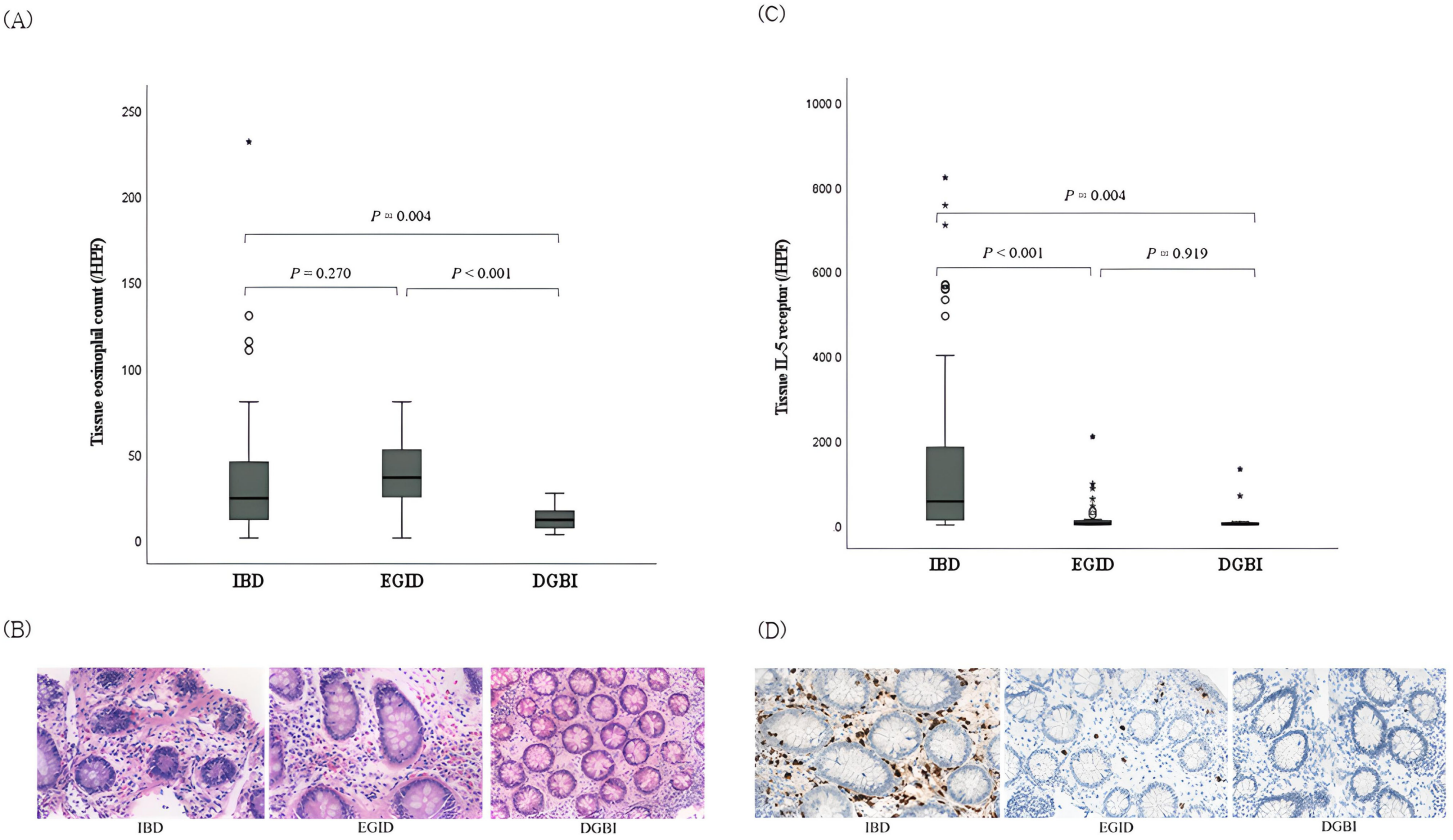
Comparison of tissue eosinophil counts and interleukin-5 receptor-α subunit expression in the colonic mucosa among pediatric patients with IBD, EGID, and DGBI. **(A)** Tissue eosinophil counts and **(B)** corresponding histopathologic findings in each disease; hematoxylin-eosin stain, magnification × 40. Significant differences were observed among the three groups (*P* = 0.003). **(C)** IL-5RA expressions and **(D)** corresponding histopathologic findings in each disease; immunohistochemical staining with anti-IL-5RA antibody, magnification × 40. Significant differences were observed among the three groups (*P <*0.001). IBD, inflammatory bowel disease; EGID, eosinophilic gastrointestinal disease; DGBI, disorder of gut–brain interaction; IL-5RA, interleukin-5 receptor-α subunit.

As an average of two slides were produced per patient for histological analysis, 99 slides for IBD (46 for CD, 41 for UC, and 12 for IBD-U), 41 for EGID, and 20 for DGBI were analyzed. When tissue eosinophil counts were compared among the IBD, EGID, and DGBI groups, a significant difference was observed, with medians (range) of 24 (1–231), 36 (1–80), and 11.5 (3–27), respectively (P=0.003). Both IBD and EGID showed significant differences from DGBI (P=0.004 and P<0.001, respectively), whereas no significant difference was observed between IBD and EGID (P=0.270) (**Figures 1A, B**).

The density of IL-5RA-expressing eosinophils was also significantly different among the IBD, EGID, and DGBI groups, with medians (range) of 141.6 (0.2–820.0), 3.0 (0.2–208.0), and 1.8 (0.2–131.4), respectively (P<0.001). Unlike the tissue eosinophil count, eosinophils in IBD expressed significantly higher IL-5RA levels than those in EGID (P<0.001; **Figures 1C, D**).

### 3.3 IBD-subtype analysis of tissue eosinophil count and IL-5RA expression

In the IBD-subtype analysis, a significant difference was observed in tissue eosinophil counts among the CD, UC, and IBD-U groups, with medians (range) of 19.5 (1–70), 35 (2–231), and 29 (2–80), respectively (P=0.020). In the two subtype analyses, tissue eosinophil counts were significantly different only between the CD and UC groups (P=0.006; **Supplementary Figures A, B**). However, the density of IL-5RA-expressing eosinophils was not significantly different among the three IBD subtypes, with medians (range) of 15.5 (0.2–708), 101.2 (0.4–755), and 127.3 (40.8–820) for CD, UC, and IBD-U, respectively (P=0.155; **Supplementary Figures C, D**).

### 3.4 Correlation between tissue IL-5RA and clinical and inflammatory parameters

On examining the correlation between IL-5RA-expressing eosinophils and tissue eosinophil count, no significant correlation was observed in the IBD, EGID, or DGBI group (all P>0.05). On analyzing the correlation between IL-5RA-expressing tissue eosinophils and clinical and laboratory findings indicative of inflammation, tissue IL-5RA expression was significantly correlated with inflammatory parameters in IBD: positively correlated with platelets, ESR, and hsCRP and negatively correlated with hemoglobin and albumin. In each IBD subgroup, IL-5RA expression was positively correlated with PCDAI, platelet count, ESR, hsCRP, and fecal calprotectin and negatively correlated with hemoglobin and albumin in CD; it was negatively correlated with peripheral eosinophil count and fecal calprotectin in UC and negatively correlated with hemoglobin in IBD-U (**Table 3**). Meanwhile, EGID and DGBI did not show significant correlations with clinical or inflammatory parameters (all P>0.05).

**Table 3:** Correlation between IL-5 receptor α and clinical or laboratory parameters in each subtype of pediatric inflammatory bowel disease.

Variable	IBD (n=50)		CD (n=23)		UC (n=21)		IBD-U (n=6)	
	r	P-value	r	P-value	r	P-value	r	P-value
PCDAI or PUCAI, median (range)			0.740	**0.001**	-0.078	0.735	PCDAI 0.319 PUCAI 0.429	0.538 0.397
Laboratory findings, mean ± SD								
Hemoglobin (g/dL)	-0.358	**0.011**	-0.490	**0.018**	-0.202	0.380	-0.829	0.042
Platelets (/μL)	0.348	**0.013**	0.446	**0.033**	0.285	0.211	0.371	0.468
Peripheral eosinophil count (/μL)	-0.068	0.641	0.154	0.484	-0.625	0.002	0.413	0.787
ESR (mm/hr)	0.380	**0.006**	0.717	**<0.001**	0.150	0.516	0.143	0.787
hsCRP (mg/dL)	0.374	**0.007**	0.734	**<0.001**	0.187	0.416	0.714	0.111
Serum albumin (g/dL)	-0.467	**<0.001**	-0.613	**0.002**	-0.275	0.227	0.086	0.872
Fecal calprotectin (mg/kg)	0.228	0.116	0.483	**0.020**	-0.446	0.049	0.029	0.957

*P*-values of < 0.05 indicate statistical significance. *P*–values indicating statistical significance highlight in bold for clarity.

IL-5, interleukin-5; IBD, inflammatory bowel disease; CD, Crohn’s disease; UC, ulcerative colitis; IBD-U, inflammatory bowel disease-unclassified; PCDAI, pediatric Crohn’s disease activity index; PUCAI, pediatric ulcerative colitis activity index; SD, standard deviation; ESR, erythrocyte sedimentation rate; hsCRP, highly sensitive C-reactive protein.

### 3.5 Analysis of tissue IL-5RA expression according to IBD severity

**Figure 2:**
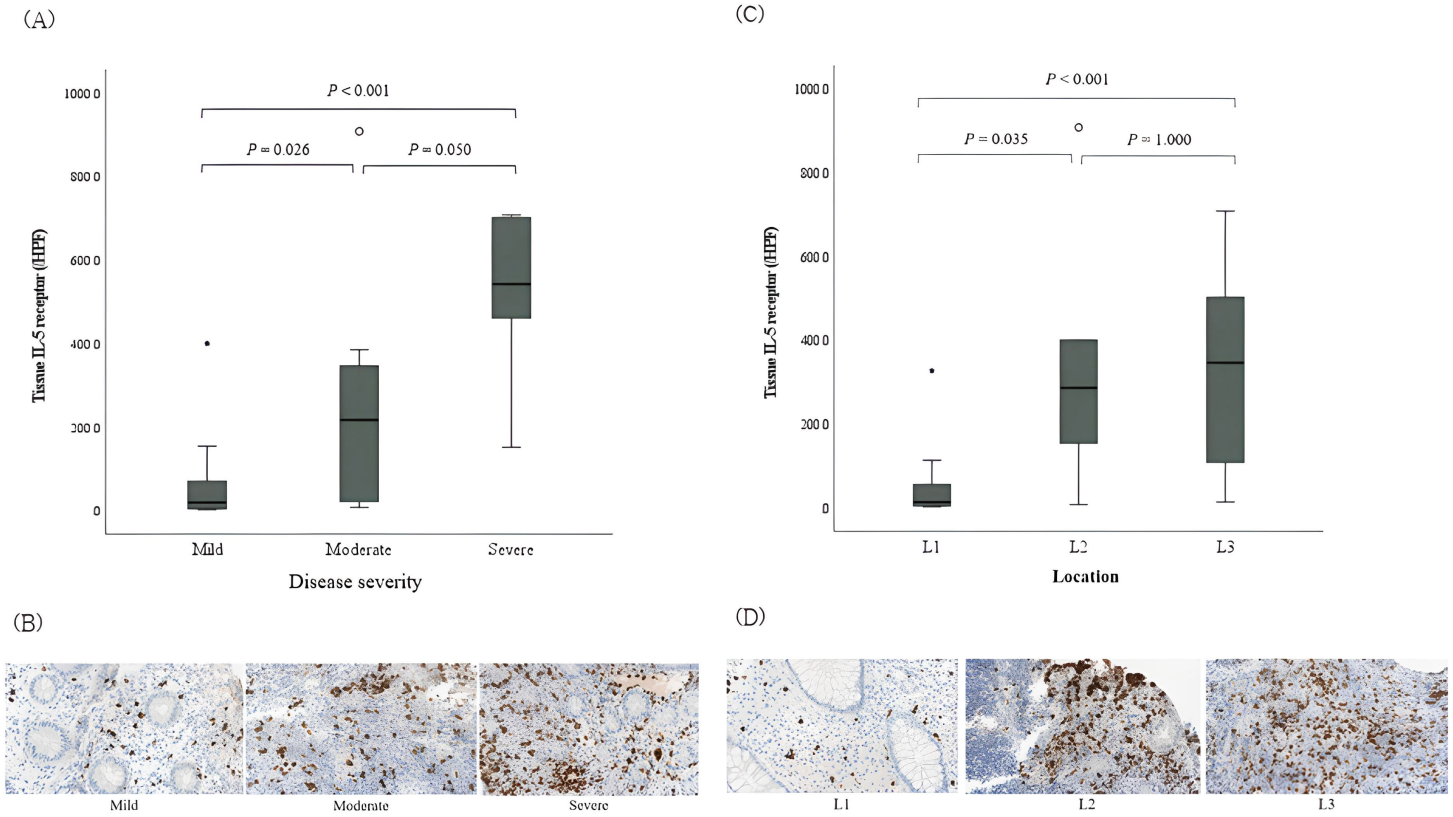
Tissue expression of interleukin-5 receptor-α subunit on eosinophils in pediatric Crohn’s disease according to the disease activity. **(A)** IL-5RA expressions according to the disease severity based on the PCDAI in CD. **(B)** Histopathologic findings of IL-5RA expressions; immunohistochemical staining with anti-IL-5RA antibody, magnification × 40. Significant differences were observed among the three groups (*P* = 0.002). **(C)** IL-5RA expressions according to the location of invaded segment. **(D)** Histopathologic findings of IL-5RA expressions; immunohistochemical staining with anti-IL-5RA antibody, magnification × 40. Significant differences were observed among the three groups (*P* = 0.004). L1, ileal with or without cecal invasion; L2, colonic invasion; L3, ileocolonic invasion; IL-5RA, interleukin-5 receptor-α subunit; PCDAI, pediatric Crohn’s disease activity index; CD, Crohn’s disease.

On analyzing IL-5RA-expressing eosinophils according to IBD severity, IL-5RA expression increased in proportion to disease severity based on the PCDAI in CD (P=0.002; **Figures 2A, B**). On examining CD according to the affected segment, a significant IL-5RA expression increase was observed in the colonic mucosa in cases with large bowel invasion compared to that in those with small bowel invasion (P=0.004; **Figures 2C, D**). However, no significant differences were observed between the cases with and without structuring or penetrating and perianal lesions. Conversely, in UC, no significant differences were observed in IL-5RA expression depending on the extent and severity according to the pediatric ulcerative colitis activity index and Paris classification.

According to the endoscopic severity, the expression of IL-5RA increased in proportion to disease severity based on the SES-CD, (P=0.023) also indicating a positive correlation between SES-CD and IL-5RA expression (r=0.635, P=0.001; **Table 4**). On the other hand, no statistically significant difference was observed between the Mayo score, endoscopic severity score in UC, and IL-5RA expression in UC.

**Table 4:** Analysis of tissue IL-5 receptor-α subunit expression according to the endoscopic severity of inflammatory bowel disease.

Crohn’s disease				*P*-value	*P*-value (mild–moderate)	*P*-value (mild–severe)	*P*-value (moderate–severe)
SES-CD	Mild (n=12)	Moderate (n=4)	Severe (n=7)				
	10.3 (3.0–400.0)	170.8 (28.4–708.0)	346.2 (13.8–701.0)	0.023	0.058	0.013	1.000
Ulcerative colitis							
Mayo	Mild (n=6)	Moderate (n=7)	Severe (n=8)				
	72.4 (2.4–574.2)	234.8 (53.4–578.6)		0.313			

*P*-values of < 0.05 indicate statistical significance.

IL-5, interleukin-5; SES-CD, simple endoscopic score for Crohn’s disease.

### 3.6 Diagnostic accuracy of IL-5RA in pediatric IBD

**Figure 3:**
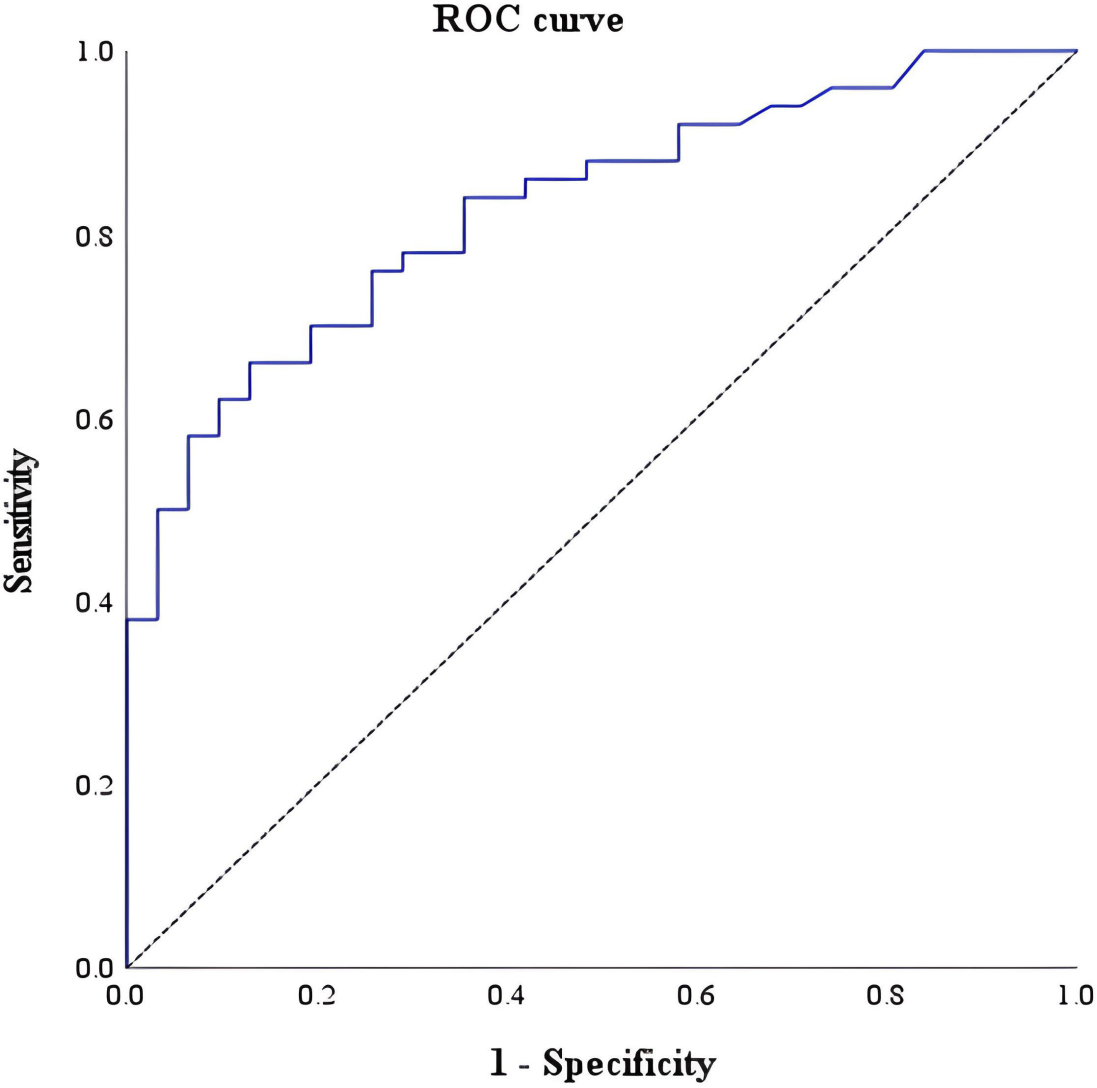
Receiver-operating characteristic curve of tissue interleukin-5 receptor-α subunit for differentiating IBD from EGID and DGBI, with a cut-off of 69.4 per high-power field.

In the receiver-operating characteristic curve analysis of the density of IL-5RA-expressing eosinophils among IBD, EGID, and DGBI, the optimal cut-off value was 69.4/high-power field, with a sensitivity of 64.3%, specificity of 90%, positive predictive value 86.5%, and negative predictive value 71.6%, revealing an AUROC for IL-5RA density of 0.825 (95% confidence interval: 0.733–0.918; **Figure 3**).

## 4 Discussion

To the best of our knowledge, this is the first study to identify IL-5RA expressed on eosinophils of the GI tract in IBD compared to that in EGID and DGBI. The present study demonstrates a potential role for IL-5 expression in IBD development beyond the presence of tissue eosinophils.

Although tissue eosinophil infiltration in the GI tract was not significantly different between IBD and EGID, tissue IL-5RA-expressing eosinophils were significantly higher in IBD than in EGID, with no difference between EGID and DGBI. These findings suggest that IL-5RA is not universally expressed in all tissue eosinophils but is elevated in inflamed tissues in early stages of active IBD. Previous studies on tissue eosinophilia in IBD have predominantly focused on UC, whereas CD has been reported to have limited association with eosinophils (9, 29–31). Similarly, in this study, tissue eosinophilia was more frequent in UC than in CD. However, in our study, no significant difference was observed in the density of IL-5RA-expressing eosinophils among patients with CD, UC, and IBD-U. This also suggests that IL-5RA expression is not only related to tissue eosinophil accumulation, but also to active inflammation in IBD. Therefore, this elevation in IL-5RA expression in tissue eosinophils may indicate a specific relationship between IL-5RA-expressing eosinophils and IBD, highlighting their potential role in IBD pathogenesis.

Furthermore, in our study, no correlation was observed between the tissue eosinophil count and IL-5RA expression in any of the three GI diseases. Additionally, allergy-related factors, such as peripheral eosinophil count, serum immunoglobulin E and eosinophil cationic protein levels, and allergy history, were not significantly correlated with IL-5RA expression in any group. This suggests that IL-5RA expression in the GI tract mucosa is not simply associated with increased eosinophils related to the allergic response, but is more likely to be significantly expressed in eosinophils that are elevated because of inflammation.

Several laboratory markers indicative of local or systemic inflammation were significantly correlated with IL-5RA expression in IBD. In CD, the positive correlation between IL-5RA-expressing eosinophils and some laboratory findings indicative of inflammation and disease severity and negative correlation between IL-5RA-expressing eosinophils and both hemoglobin and albumin, which worsen with increasing disease severity, also indicate that IL-5RA-expressing eosinophils are good markers of active inflammation in CD. Conversely, IL-5RA-expressing eosinophils were not significantly correlated with laboratory findings indicative of inflammation in UC. UC typically presents with lower levels of inflammatory markers, such as white blood cells, platelet count, ESR, and hsCRP, compared to CD, and albumin levels are relatively normal (32). Consequently, these hematological markers may not adequately reflect active inflammation in UC, rendering the correlation with IL-5RA-expressing eosinophils non-significant. In contrast, studies have shown that peripheral eosinophilia reflects disease severity in UC (30, 31, 33, 34). The positive correlation between IL-5RA-expressing eosinophils and peripheral eosinophils in our study suggests that IL-5RA-expressing eosinophils also reflect active inflammation in UC. However, IL-5RA expression negatively correlated with fecal calprotectin, reflecting the endoscopic severity of UC (35). In our study, among the 21 patients with UC, 6 (28.6%) had severe UC, while 15 (71.4%) did not, which may have limited the ability to reflect fecal calprotectin levels.

In CD, IL-5RA expression also differed depending on the location of the invasive segment. For the ileal with or without cecal invasion (L1) group, one tissue sample was collected from the cecum with a lesion and the other from a region without a grossly active lesion. IL-5RA expression, which reflects active inflammation, was lower in the L1 group than in the colonic invasion (L2) and ileocolonic invasion (L3) groups, indicating active inflammation in the colon. Therefore, the lower IL-5RA-expressing eosinophils in the L1 group were consistent with less inflammation than those in the L2 and L3 groups. Furthermore, in CD, higher endoscopic severity was associated with greater IL-5RA expression. A positive correlation between endoscopic severity and IL-5RA expression suggests that, unlike in UC, IL-5RA expression in CD may reflect clinical and endoscopic severities.

CD is primarily associated with Th1 immunity, whereas UC is more closely linked to Th2 immunity (14). Especially in CD, eosinophils are not the primary immune cells driving disease pathogenesis. Instead, as disease severity increases, various immune cells are likely to contribute to inflammation, potentially altering the activation state of eosinophils. Additionally, previous studies have suggested that eosinophils are associated with fibrosis during the chronic phase of inflammation (7). Since CD is characterized by transmural involvement, in cases with severe transmural invasion, eosinophils may plausibly play a more significant role. This study hypothesizes that IL-5RA expression is more prominent in active eosinophils, potentially explaining the differences in IL-5RA expression patterns observed between UC and CD. The differential roles of eosinophils and IL-5RA in these two IBD subtypes warrant further investigation to elucidate their contributions to disease pathogenesis.

IL-5 and its receptor appear to contribute to the pathogenesis of IBD through diverse mechanisms. A previous study utilizing dextran sulfate sodium (DSS) -induced colitis models demonstrated that IL-5 antagonists effectively inhibit IL-5/IL-5RA signaling pathway, leading to the suppression of STAT5 activity and reductions in IL-1β and caspase-1 levels (36), contributing to the development of IBD by promoting intestinal inflammation and tissue damage (37). Another study employed a combination of an IL-2/IL-2 agonist immunocomplex with IL-5RA antagonist in the DSS colitis model, showing superior therapeutic efficacy compared to using the blocking IL-2/IL-2 agonist immunocomplex alone. This combination effectively inhibited eosinophil activation induced by IL-2, thereby enhancing the treatment outcomes (38). Collectively, these studies underscore the critical role of eosinophil and IL-5RA signaling in IBD pathogenesis, with IL-5RA antagonist demonstrating significant potential in improving disease outcomes in DSS-induced colitis models. Based on these preclinical findings, this study is the first study to quantify IL-5RA expression in tissue eosinophils in human IBD patients, confirming its upregulation and directly implicating its involvement in IBD pathophysiology.

Additionally, in the JAK-STAT pathway, IL-5 activates JAK2, which in turn phosphorylates downstream STAT3 and STAT5. Notably, STAT3, known to play a role in IBD pathogenesis through IL-23 and IL-6 activation, may also be activated by IL-5. The aforementioned study (36) proposed the potential involvement of STAT5 in the pathogenesis of IBD. However, the precise mechanisms linking the IL-5 specific JAK-STAT pathway to IBD pathogenesis remain unclear. The two studies referenced above demonstrated that IL-5RA antagonists ameliorate colitis through distinct mechanisms. Thus, eosinophil and IL-5/IL-5RA could be implicated in IBD pathogenesis through its interaction with various cytokines and inflammatory pathways.

When plotting the receiver-operating characteristic curve, among the various laboratory results collected and analyzed in this study, IL-5RA revealed the second widest AUROC, followed by fecal calprotectin (AUROC: 0.912, 95% confidence interval: 0.845–0.980). In addition to fecal calprotectin, IL-5RA expression may serve as a valuable indicator with high sensitivity for diagnosing IBD.

This study has some limitations. It was conducted retrospectively with tissues obtained from endoscopic biopsy specimens collected for diagnostic purposes at initial diagnosis, which may introduce selection bias. Although we aimed to match the segments of the analyzed tissues, limitations existed in matching the segments because the affected segments were different for each patient. Additionally, because of the small sample size, limitations exist in matching and analyzing disease characteristics, such as extent and behavior, within the IBD subgroups. Therefore, further studies with larger cohorts are warranted.

A key strength of this study is the investigation of the distinctive IL-5 receptor of eosinophils in IBD by performing immunohistochemistry targeting specific cytokine receptors that, to our knowledge, have not been previously studied in granulocytes, particularly eosinophils, in tissues from patients with treatment naïve active IBD. This approach allowed the identification of specific eosinophilic receptors in active IBD. Additionally, this study expands the scope by proposing that eosinophils, particularly IL-5RA, may be associated not only with disease severity in UC, but also in CD. Furthermore, it suggests a potential relationship between IL-5RA expression and the anatomical localization of disease involvement in both conditions, underscoring its relevance to disease pathophysiology.

Recent studies have shown that biologics targeting IL-5 or IL-5RA can be adapted to treat various allergic diseases, including eosinophilic esophagitis (11, 39). Based on our study, further research may be needed to demonstrate the role of eosinophils in terms of IL-5 and its receptor that is involved in IBD. This might lead to the possibility of a targeted therapy utilizing IL-5RA as a target in the IBD phenotype, in which IL-5RA predominates.

In conclusion, the expression of IL-5RA in tissue eosinophils, a hallmark of the treatment-naïve early active state of IBD, particularly CD, may also reflect disease activity and mucosal lesions. Therefore, this study is a step closer to demonstrating the important role of tissue eosinophils in IBD pathogenesis.

## Data Availability

The raw data supporting the conclusions of this article will be made available by the authors, without undue reservation.
