# Supplementary figures and images for: Remarkable increase in Interleukin-5 receptor expression beyond tissue eosinophils in inflammatory bowel disease
Source: Front Immunol. 2025 May 22;16:1589421. doi: 10.3389/fimmu.2025.1589421 (PMC12137258; doi:10.3389/fimmu.2025.1589421)

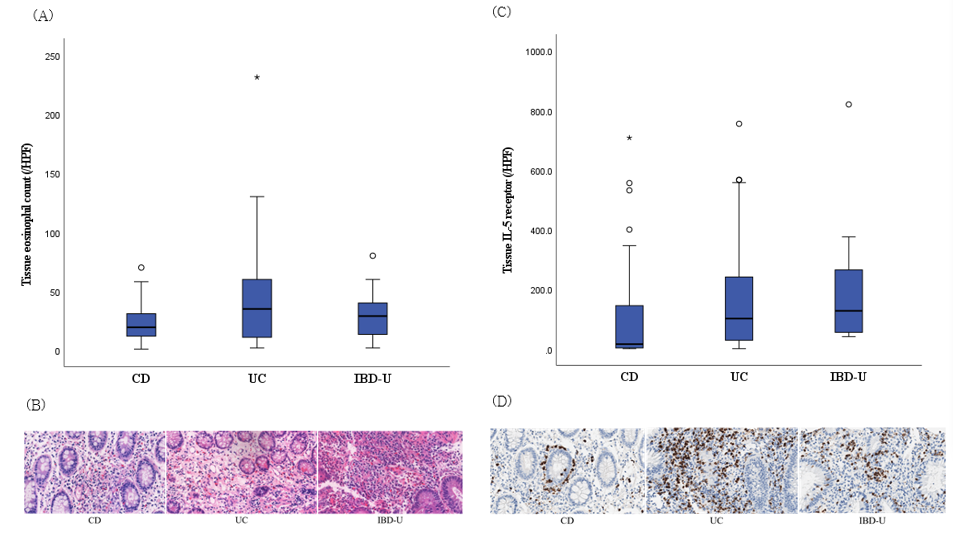

Supplement: Supplementary file 1 [file Image1.tif]
